# Ageing prolongs inflammatory marker expression in regenerating rat skeletal muscles after injury

**DOI:** 10.1186/1476-9255-8-41

**Published:** 2011-12-29

**Authors:** Chris van der Poel, Luc E Gosselin, Jonathan D Schertzer, James G Ryall, Kristy Swiderski, Meron Wondemaghen, Gordon S Lynch

**Affiliations:** 1 Basic and Clinical Myology Laboratory, Department of Physiology, The University of Melbourne, Victoria, 3010, Australia; 2 Department of Exercise and Nutrition Sciences, University of Buffalo, Buffalo, NY 14214, USA; 3 Current: Department of Human Biosciences, Faculty of Health Sciences, La Trobe University, Bundoora, 3086, VIC, Australia; 4 Current: Faculty of Health Sciences, Dept. of Biochemistry and Biomedical Sciences, Hamilton ON, L8S 4K1, Canada; 5 Current: The Laboratory of Muscle Stem Cells and Gene Regulation, National Institute of Arthritis, Musculoskeletal and Skin Diseases, National Institutes of Health (NIH), Bethesda, MD; USA; 6 Current: Faculty of Arts, Monash University, Clayton, 3800, Victoria, Australia

## Abstract

**Background:**

Some of the most serious consequences of normal ageing relate to its effects on skeletal muscle, particularly significant wasting and associated weakness, termed "sarcopenia". The underlying mechanisms of sarcopenia have yet to be elucidated completely but an altered muscle inflammatory response after injury is a likely contributing factor. In this study we investigated age-related changes in the expression of numerous inflammatory markers linked to successful muscle regeneration.

**Methods:**

Right extensor digitorum longus (EDL) muscles from young (3 month), adult (12 month) and old (24 month) male F344 rats were injected with bupivacaine hydrochloride to cause complete muscle fibre degeneration, then excised 12, 24, 36, and 72 hours later (n = 5/age group/time point). We used qRT-PCR to quantify the mRNA expression levels of the inflammatory markers TNFα, IFNγ, IL1, IL18, IL6, and CD18 as well as regenerative markers MyoD and myogenin.

**Results:**

Inflammatory markers were all increased significantly in all age groups after myotoxic injury. There was a trend for expression of inflammatory markers to be higher in uninjured muscles of old rats, especially at 72 hours post injury where the expression levels of several markers was significantly higher in old compared with young and adult rats. There was also a decrease in the expression of regenerative markers in old rats at 72 hours post injury.

**Conclusion:**

Our findings identify a prolonged inflammatory signature in injured muscles from old compared with young and adult rats together with a blunted expression of key markers of regeneration in muscles of old rats. Importantly, our findings identify potential targets for future therapeutic strategies for improving the regenerative capacity of skeletal muscle during ageing.

## Introduction

Skeletal muscle regeneration is a complex process composed of three stages: (1) myofibre degeneration; (2) inflammation; and (3) myofibre regeneration and involves the activation of quiescent satellite cells which, through the processes of proliferation and differentiation, participate in the reconstitution of damaged tissues [1,2]. Successful skeletal muscle regeneration after injury requires a carefully regulated inflammatory response to remove cell debris and initiate the activation of the normally quiescent satellite cells [3]. It is widely accepted that inflammation is a natural response to acute skeletal muscle injury as blocking inflammatory cell function by various methods has been demonstrated to result in poor muscle regeneration [4-6]. However, what constitutes an appropriate inflammatory response versus a damaging response remains poorly understood.

During the early inflammatory response to injury, the most abundant immune cell types at the injury site are neutrophils which play two roles. Firstly, they play a phagocytic function to clear the injury of necrotic cells, and secondly they are thought to enhance the inflammatory response via the release of pro-inflammatory cytokines such as interleukin-6 (IL-6) and tumor necrosis factor-alpha (TNFα) [2]. However, there is also evidence to suggest that neutrophils may subsequently cause secondary damage to the muscle as neutrophil depletion and/or manipulation of neutrophil function have been demonstrated to reduce muscle damage [2]. Inflammatory cell infiltration, including both neutrophils and macrophages, is associated with an increase in a number of pro-inflammatory cytokines such as IL-1β, tumor necrosis factor (TNF), and transforming growth factor beta-1 (TGF-β1). A secondary phase of the inflammatory response is indicated by an influx of macrophages, which appears to coincide with a decline in neutrophil numbers. Macrophages play a role in the removal of necrotic debris and produce soluble factors that promote regeneration [2]. Previous studies in rats have revealed that 2-3 days after lengthening contraction-induced injury, there is a temporary increase in ED^1+ ^(infiltrating) macrophage content in damaged muscles followed by a transient rise in ED^2+ ^(resident) macrophages, which are associated with muscle regeneration and repair [7,8].

In mammals, ageing is associated with a progressive decline in skeletal muscle mass and function, called 'sarcopenia' [9]. Muscles of old animals are more susceptible to injury, they regenerate poorly, and functional recovery remains incomplete [10-13]. Cycles of damage and less-than-complete repair (due to decreases in circulating anabolic hormones and growth factors) cause muscle atrophy and weakness with age. Furthermore, chronic low-grade systemic inflammation and prolonged inflammatory responses to infection and (muscle) injury are thought to contribute to decreased muscle protein synthesis and reduced regenerative capacity in the elderly, given that increased amounts of circulating pro-inflammatory cytokines, such as IL-6 and TNF-α, have been associated with muscle wasting [13].

The impact of ageing on the inflammatory/cytokine response of human skeletal muscles after injury remains poorly understood, but there is strong evidence indicating the importance of inflammatory factors in the onset and progression of age-related muscle wasting [14,15]. Comparison of the 'molecular signature' between young and old males using microarrays, revealed an increased expression of several inflammatory and apoptotic genes [14]. Age-related differences in the expression levels of TNF-α, IL-6, CD18, and TGF-β1 have also been demonstrated after an acute bout of eccentric exercise [15]. However, in that study, both young and old subjects were exercised at a similar percentage of VO_2max_, and so given that older subjects have a lower functional capacity, the mechanical forces placed on the muscles may have differed significantly between the groups, which could have contributed to the different amounts of muscle damage and resulting cytokine response.

Work by Carlson and Faulkner and their colleagues over several decades showed that the recovery from skeletal muscle injury was prolonged in aged rats [16]. An altered inflammatory response (with respect to timing or concentration) may affect the cellular processes required for optimal muscle remodeling (i.e. degeneration and regeneration). More recently, the inflammatory responses to whole muscle grafting were characterized in young, adult and old mice [17]. This form of injury which involves a loss of vascular supply to the muscle resulted in delayed regeneration in old compared with younger mice, a finding attributed to a less than vigorous inflammatory response by old host mice and a less potent chemotactic stimulus produced by the damaged muscles of old mice [17].

In the present study, we tested the hypothesis that the inflammatory response is dysregulated with ageing in skeletal muscle damaged by the myotoxin, bupivacaine. This model of injury was chosen since it causes rapid and complete degeneration of the fibres but leaves existing vasculature, nerves, satellite cells, and basal lamina intact [18,19]. It is a reliable and reproducible form of skeletal muscle injury that avoids potential confounding factors such as damage to nerves, vascular supply and extracellular matrix and unequal amounts of muscle fibre damage and regeneration, allowing for examination of events concerned solely with muscle fibre regeneration.

## Materials and methods

### Animals

All procedures were approved by the Animal Ethics Committee of The University of Melbourne and were conducted in accordance with the Australian code of practice for the care and use of animals for scientific purposes as stipulated by the National Health and Medical Research Council of Australia. Male Fischer 344 rats aged 3 month (young), 12 month (adult), and 24 month (old) were obtained from the National Institute on Aging colony maintained by Harlan Sprague Dawley (Indianapolis, IN). All animals were housed in a pathogen-free environment in standard cages and had free access to food and water. The rats were housed under an artificial light: dark cycle with light between 0600 and 1800 hour.

### Injury protocol

Animals were anaesthetised with sodium pentobarbitone (Nembutal, Rhone Merieux, Pinkenba, QLD, Australia, 60 mg/kg, *i.p*). The right hindlimb was shaved and a small portion of the extensor digitorum longus (EDL) muscle surgically exposed and injected with 0.5% bupivacaine hydrochloride (bupivacaine) (~500 μl, Marcain, Astra, North Ryde, NSW, Australia) using a 29-gauge fixed needle. Several injections were made in the distal, proximal, and midbelly regions of each muscle. This volume was equivalent to, or exceeded, the maximum volume of bupivacaine that each EDL muscle could hold and that caused degeneration of all fibres [19,20]. The EDL muscle of the left hindlimb served as the non-injected control in all cases. These muscles were not injected with saline vehicle, since we and others have shown that injection of saline alone does not affect the morphological, structural, or functional characteristics of skeletal muscles [18,19]. After the intramuscular injection, the skin incision was closed with Michel clips (Aesculap, Tuttlingen, Germany). After the surgical procedure, rats were randomly assigned to testing at 12, 24, 36, 72 hours or 7 days after myotoxic injury (n = 5/age group/time point). After the specified duration of recovery, rats were anaesthetised deeply with pentobarbital sodium (60 mg/kg body mass *i.p*), and the EDL muscles from the left (untreated) and right (bupivacaine injected) hindlimb were surgically excised and flash-frozen in liquid nitrogen and stored at -80°C.

### RT-PCR analyses

RNA was isolated from a section of the EDL muscle (~ 15-30 mg) per the manufacturer's instructions using the RNeasy Fibrous Tissue kit (74704; Qiagen, Victoria, Australia). RNA concentration was determined by UV absorption at 260 nm, and the samples stored at -80°C. For each sample, 5 μl total RNA was reverse transcribed to cDNA using a SSIII First strand cDNA synthesis kit (Invitrogen) with the resulting cDNA diluted to 12.5 ng/μl and stored at -80°C. Real-time PCR (iQ5 Cycler detection system BioRad) was run for 1 cycle (50°C for 2 min, 95°C for 3 min) and 40 cycles (95°C for 20 s, 55°C for 30 s). Fluorescence from the incorporation of SYBR Green (SYBR green master mix; BioRad) to double-stranded DNA was measured after each repetitive cycle. All samples were run in triplicate and measurements included a no-template control (no cDNA). Primer sequences were designed for TNF α, IL-6, IL-1β, IFN γ, CD18, IL-18 (Table 1). Gene expression was quantified using a cycle threshold (CT) method, whereby the relative expression of the genes compared with resting samples was made using the expression 2^-CT ^in which the expression of each gene was normalised to the concentration of input cDNA. Myotoxic injury following a maximal injection of bupivacaine causes complete degradation of skeletal muscle fibres and therefore normalizing mRNA to a housekeeping gene is difficult. For all comparisons between samples, mRNA was normalized to the cDNA concentration as determined by Quanti-iT OliGreen ssDNA Assay Kit (Molecular Probes, Eugene, OR, USA) and expressed as mRNA relative to cDNA (arbitrary units).

**Table 1:** Forward and reverse primers for PCR analyses.

*Transcript* (Accession #)	Forward	Reverse
CD18 (NM-001037780)	TGA CAT CCC CAA CAA AGT GA	CAC TCG GCA CAG AAG ACG TA
TNF-α (NM-012675)	GCT CCC TCT CAT CAG TTC CA	TGT GGG TGA GGA GCA CAT AG
IL-1β (NM-031512)	AGG CTT CCT TGT GCA AGT GT	TGA GTG ACA CTG CCT TCC TG
IL-6 (NM-012589)	TGT GCA ATG GCA ATT CTG AT	GAG CAT TGG AAG TTG GGG TA
IL-18 (NM-019165)	ATA TCG ACC GAA CAG CCA AC	ATC CCC ATT TTC ATC CTT CC
IFN-γ (NM-138880)	GCC CTC TCT GGC TGT TAC TG	CCA AGA GGA GGC TCT TTC CT
Myogenin (M-24393)	TGG TCC CAA CCC AGG AGA TCA TTT	ACA TAT CCT CCA CCG TGA TGC TGT
MyoD (M-84176)	CTA CAG CGG CGA CTC AGA CG	TTG GGG CCG GAT GTA GGA

All sequences are read from 5' to 3'. Corresponding accession numbers for the rat (*Rattus norvegicus*) are listed below the gene.

### Statistical analyses

All data were analyzed using a two-way analysis of variance between groups (1-way or 2-way ANOVA where appropriate) for age versus time with post-hoc (Bonferroni's) analysis. A *P *value less than 0.05 was considered statistically significant. All data are presented as mean ± SEM, unless otherwise stated.

## Results

### Inflammatory cytokine mRNA expression

**Figure 1:**
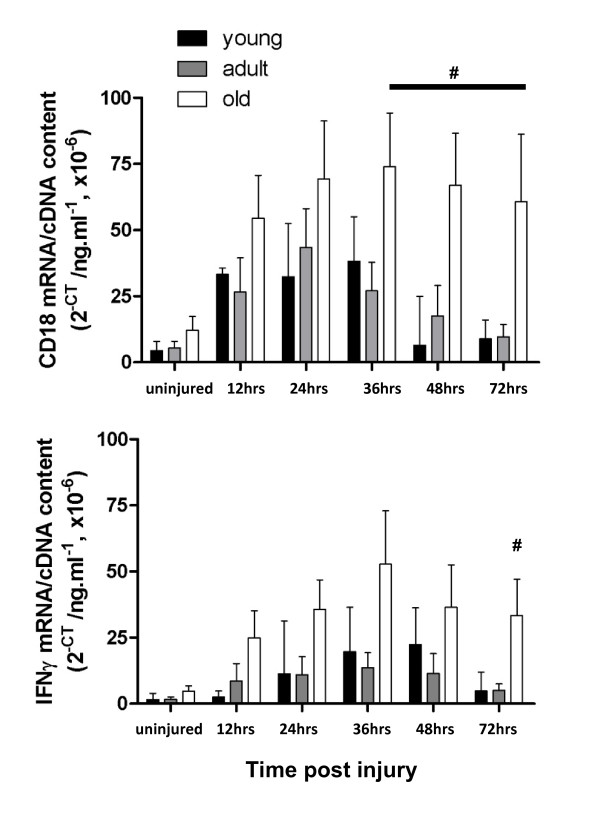
**Total mRNA transcript levels of CD18 (A) and IFNγ (B) in the injured EDL muscles of all age groups**. # represents significant difference between old compared with young and adult rats (*P *< 0.05).

CD18, a cell surface adhesion molecule that is critical for the accumulation of neutrophils to damaged skeletal muscle [21], and IFNγ, which is best described as a macrophage activating protein, are important initiators of the inflammatory response in skeletal muscle. Figure 1 shows the total mRNA transcript levels of CD18 (upper panel) and IFNγ (lower panel) in the EDL muscles of all age groups both pre and post injury. In young and adult rats, both CD18 and IFNγ levels were low in uninjured muscle, rapidly increased within 12 hours post injury, and had begun to decrease to basal levels by 72 hours post injury. CD18 and IFNγ mRNA levels were not significantly different in uninjured muscles from old compared with young and adult rats. Following injury, both CD18 and IFNγ levels increased within 12 hours as expected, but remained higher at least to 72 hours post injury compared with injured muscles of young and adult rats. A 2-way ANOVA srevealed a significant effect for both age and time for CD18 (age and time effect; *P *< 0.0001) and IFNγ (age and time effect; *P *< 0.0001). However there was only a significant interaction between age and time for CD18 (P = 0.048).

**Figure 2:**
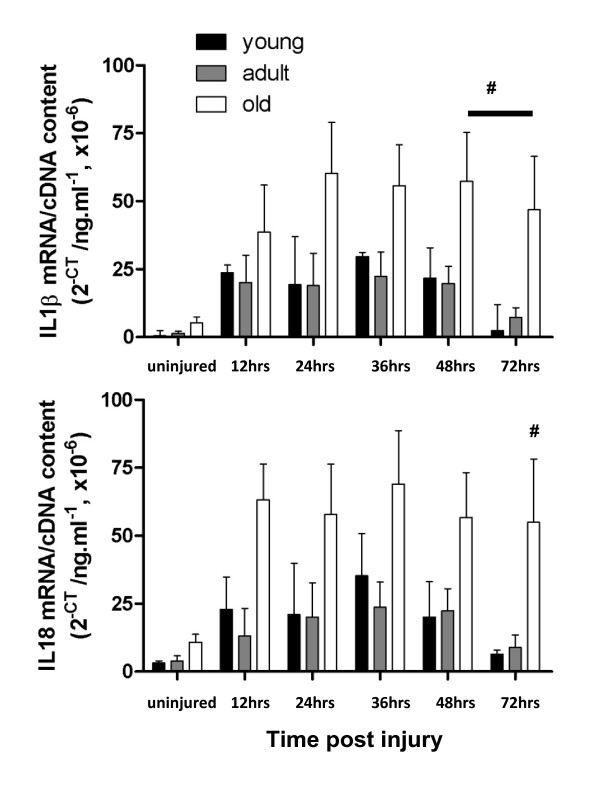
**mRNA transcript levels of IL1β and IL18 in injured and uninjured muscles from young, adult old rats**. # represents significant difference between old compared with young and adult rats (*P *< 0.05).

IL1β, which is released by damaged muscle to attract neutrophils and macrophages, and IL18, which is released by macrophages, were also elevated in the muscles from uninjured old rats (Figure 2 upper and lower panel, respectively). As observed for CD18 and IFNγ, both IL1β and IL18 levels increased rapidly in all three age groups after injury but remained elevated in the old rats at 48 and 72 hours post injury for IL1β and only at 72 hours post injury for IL18. For both IL1β and IL18 there was a significant age and time effect (*P *< 0.0001) but only a significant interaction between them for IL1β (*P *< 0.0006).

**Figure 3:**
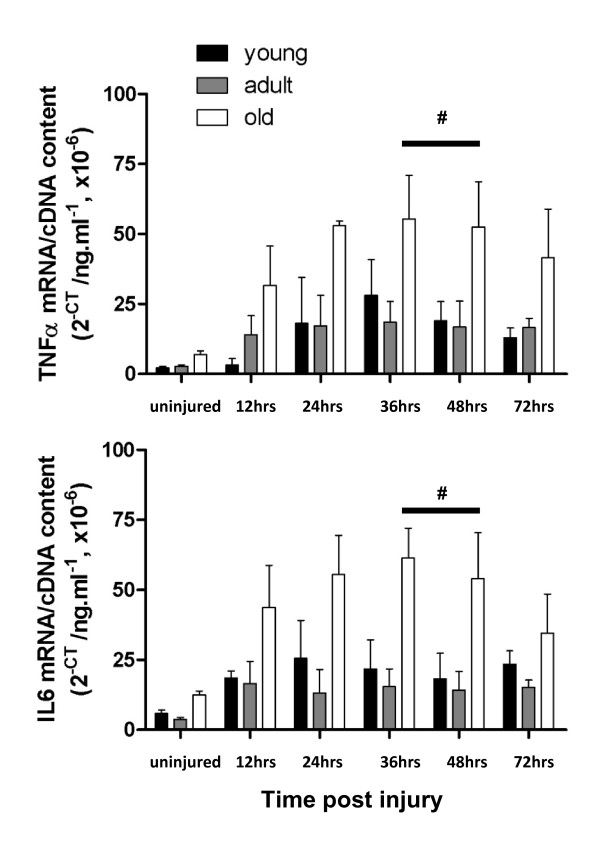
**mRNA transcript levels of TNFα in muscles from young, adult old rats at 12, 24, 36, 48 and 72 hours post-injury**. # represents significant difference between old compared with young and adult rats (*P *< 0.05).

TNFα is released by macrophages and is involved in a number of different processes including pro-inflammatory and cell-death pathways. At 36, and 48 hours post-injury there was a significantly higher transcript level of TNFα mRNA in the muscles from old rats compared with those from young and adult rats (Figure 3, upper panel). Total transcript levels of IL6, which is released by damaged muscle and plays a role in attracting immune cells to damaged areas, were significantly greater in the muscles from old compared with muscles from adult and young rats at 36 and 48 hours post injury (Figure 3, lower panel). There was a significant age and time effect for both TNFα and IL6 post-injury (*P *< 0.0001), but there was no interaction between them (TNFα; *P *= 0.3046 and IL6; *P *= 0.0919). Together these data indicate that the dysregulation of the inflammatory response is more pronounced and prolonged in EDL muscles of old rats after myotoxic injury.

### Myogenic regulatory factor mRNA expression

**Figure 4:**
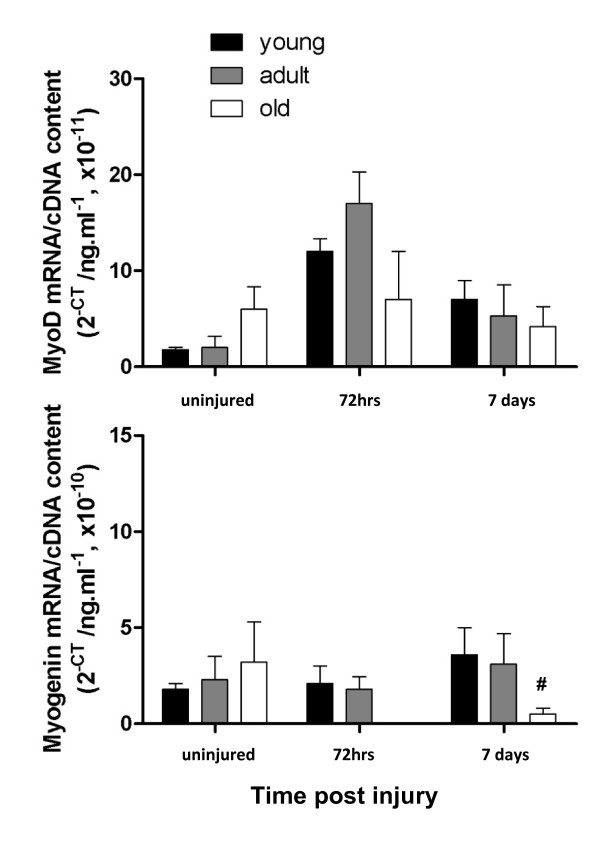
**Total MyoD (A) mRNA expression in uninjured EDL muscles higher in old rats compared with young and adult rats**. Ø represents significant difference between old compared with young (*P *< 0.05).

MyoD is one of the earliest markers of myogenic differentiation and it plays a key role in the early stages of regeneration, including satellite cell activation and promotion of differentiation. Therefore, MyoD expression levels indicate whether the regeneration process has been initiated. In young and adult rats, MyoD mRNA expression was increased at 72 hours and had begun to decrease by 7 days post-injury. In old rats, MyoD expression was higher in uninjured muscle when compared to young and adult rats, however, expression failed to increase at 72 hours post injury (Figure 4, upper panel). There was no significant age effect on MyoD expression (*P *= 0.2168) but there was a significant time effect (*P *> 0.0001) and a significant interaction (*P *= 0.0028) between the responses.

Myogenin is a transcription factor important during the later stages of muscle fibre regeneration and is involved in the activation of myosin heavy chains, as well as in the fusion and maturation of new muscle fibres. In uninjured muscles, there was no difference in myogenin expression in EDL muscles of rats regardless of age. However, following myotoxic injury, myogenin transcript levels were expressed differently from those observed for MyoD such that at 72 hours post injury there was no significant difference between myogenin levels in muscles from young and adult and there was no detectable signal in the muscles from old rats (Figure 4, lower panel). By 7 days post-injury, myogenin levels had increased by 90% in the muscles of young and adult rats, while in the muscles from old rats myogenin was detectable but at significantly lower levels than in muscles of young and adult rats (1 way ANOVA, *P *< 0.05; Figure 4, lower panel). There was no significant age or time effect on myogenin expression (*P *= 0.5245 and 0.9162, respectively), and no significant interaction between age groups (*P *= 0.052).

## Discussion

Although a number of different hypotheses have been proposed to explain the cause of the age-related impairment in skeletal muscle regeneration, the exact mechanisms remain elusive. In this study, we provide new evidence for an extended inflammatory signature in injured muscles from old compared with young and adult rats; and a blunted expression of key markers of regeneration in injured muscles from old rats. These new findings add considerably to our understanding of age-related changes to mammalian skeletal muscle and the regenerative responses after injury.

There is considerable evidence for altered inflammatory cytokine signaling in skeletal muscles with ageing [15,17,22-25]. The plasma concentration of cytokines that play central roles in the early stages of the pro-inflammatory response, such as IL-6 and TNFα, are increased in elderly individuals, and these are correlated significantly with an age-associated increase in adipose [26] and a concomitant decrease in muscle strength [23,26]. This observation was different from that of Hamada and colleagues who reported no difference in TNFα levels between active young and old individuals before exercise, although adipose content was not taken into account in this study and could explain the disparity in the observations reported [15]. The findings of the present study are consistent with an increase in basal TNFα mRNA expression in uninjured contralateral EDL muscles [23,26] and support strongly the hypothesis that ageing is associated with an increase in cytokine levels. Other studies have shown decreased levels at rest and impaired responses to exercise for mRNAs for factors implicated in muscle growth and development [27,28]. In young and old men subjected to resistance exercise, expression of IL-1β was not different between groups at baseline but increased within 24 hours of the exercise bout only in young subjects. These findings and those from related studies suggest that pro- and anti-inflammatory cytokine responses to damaging exercise are impaired with ageing [28,29]. Yet despite studies suggesting a link between inflammation and ageing, the direct effect of inflammation on muscle mass and strength has only been demonstrated recently. In a model of local muscle inflammation where casein was injected directly into soleus muscles of rats, there was a significant reduction in muscle mass and strength [30] which was only fully restored 2 weeks after resolution of the inflammatory response. Considering the age-related increase in inflammation and reduced muscle fibre regeneration after injury, one interpretation is that the biological "switch" that regulates the inflammatory response is impaired with ageing.

Following the inflammatory response, activation of normally quiescent satellite cells is integral to the processes of repair and growth of skeletal muscle [31]. These processes are modulated by a variety of local factors, including the family of transcription factors known as myogenic regulatory factors (MRFs) (MyoD, and myogenin) [32]. Although MyoD and myogenin are barely detectable in skeletal muscles of adult animals [33], elevated mRNA of MyoD and myogenin has been demonstrated in skeletal muscles of aged animals [33,34], whereas protein levels are blunted in muscles from aged rats [35,36]. It should be noted, however, that these findings are based on basal levels or levels measured immediately after a single bout of exercise. Only one study has investigated the MRF profile in a model of senescence. Bigot and colleagues [37] showed that in the process of differentiation from myoblast to myotube, mRNA expression of a number of MRF's were reduced significantly and/or delayed in senescent human myoblasts. Activation of myogenin was delayed by 15 hours and reduced 7-fold in senescent myoblasts compared with those from young, whereas MyoD expression was 3.3-fold lower in senescent cells. Although the results from these experiments support the findings of the present study, it should be noted that they were conducted in the absence of a chronic inflammatory signal, indicating that there are other factors besides altered inflammation that also contribute to the age-related decline in muscle regenerative capacity.

It should be noted that different models of injury are likely to result in differences in muscle inflammatory responses. In the present study, we used a myotoxic model of injury where the damage was limited only to muscle fibres and not to other structures. Other models, such as whole muscle grafting [17] are likely to elicit a different inflammatory signature and MRF expression during regeneration given that the blood supply to the grafted muscle is compromised and successful revascularisation is necessary for successful muscle repair.

Our findings revealed that the altered inflammatory response was linked with a blunted muscle regenerative response in aged rats. We found that the basal levels of inflammatory cytokines are higher in muscles from old rats and the inflammatory response following myotoxic injury was prolonged in old compared with young and adult rats. These findings are consistent with the notion of aberrant inflammatory cytokine signaling in skeletal muscle ageing and that loss of the inflammatory regulation contributes to the age-related impairment in muscle regenerative capacity.

## Competing interests

The authors declare that they have no competing interests.

## Authors' contributions

CVPD, LEG, JDS, JGR and GSL conceived, designed and conducted experiments for the study. MW and KS performed experiments and helped in constructing the figures. CVDP, LEG and GSL wrote the manuscript with critical input from all the authors who read and approved the final manuscript.
